# Prevalence and Risk Factors of Hypoxemia after Coronary Artery Bypass Grafting: The Time to Change Our Conceptions

**Published:** 2019-04

**Authors:** Fardin Yousefshahi, Elham Samadi, Omalbanin Paknejad, Ali Movafegh, Khosro Barkhordari, Ehsan Bastan Hagh, Babak Dehestani

**Affiliations:** 1 *Department of Anesthesiology, Imam Khomeini Hospital Complex, Tehran University of Medical Sciences, Tehran, Iran.*; 2 *Kelvington Hospital, Kelvington, Saskatchewan, Canada.*; 3 *Department of Pulmonology, Shariati Hospital, Tehran University of Medical Sciences, Tehran, Iran.*; 4 *Department of Anesthesiology, Shariati Hospital, Tehran University of Medical Sciences, Tehran, Iran.*; 5 *Tehran Heart Center, Tehran University of Medical Sciences, Tehran, Iran.*; 6 *School of Medicine, Tehran University of Medical Sciences, Tehran, Iran.*

**Keywords:** *Risk factors*, *Hypoxia*, *Coronary artery bypass*, *Respiratory distresssyndrome, adult*

## Abstract

**Background:** Acute hypoxemia is the main characteristic of acute respiratory distress syndrome (ARDS), which is one of the most critical complications of coronary artery bypass grafting (CABG). Given the dearth of data on acute hypoxemia, we sought to determine its prevalence and risk factors among post-CABG patients.

**Methods:** This cross-sectional study was conducted on on-pump CABG patients in Tehran Heart Center in 2 consecutive months in 2012. The effects of arterial blood gas variables, age, gender, the duration of the pump and cross-clamping, the ejection fraction, the creatinine level, and the body mass index on the prevalence of hypoxemia at the cutoff points of ARDS and acute lung injury were assessed.

**Results:** Out of a total of 232 patients who remained in the study, 174 (75.0%) cases were male. The mean age was 60.60±9.42 years, and the mean body mass index was 27.15±3.93 kg/m^2^. None of the patients expired during the current admission. The ratio of partial pressure arterial oxygen to the fraction of inspired oxygen (PaO_2_/FiO_2_) 1 hour after admission to the intensive care unit (ICU), before extubation, and at 4 hours after extubation was less than 300 mmHg in 66.6%, 72.2%, and 86.6% of the patients and less than 200 mmHg in 20.8% 17.7%, and 30.2% of the patients, respectively. Among the different variables, only a heavier weight was associated with a PaO_2_/FiO_2_ ratio of less than 300 mmHg at 1 hour after ICU admission and at 4 hours after extubation (P=0.001). A rise in the cross-clamp time showed a significant association with the risk of a PaO_2_/FiO_2_ ratio of less than 200 mmHg at 4 hours after extubation (P=0.014).

**Conclusion:** This study shows that hypoxemia following CABG is very common in the first 48 postoperative hours, although it is a benign and transient event. The high prevalence may affect the accuracy of the ARDS criteria and their positive or negative predictive value.

## Introduction

Acute respiratory distress syndrome (ARDS) is a common clinical failure recognized with the injury to the alveolar epithelium and the pulmonary endothelial tissue and accompanied by pulmonary edema, resulting in acute respiratory distress.^1^ The onset of this syndrome is typically accelerated and it usually happens within only a few of hours in patients with no past history of pulmonary diseases.^2^ Currently, the most common diagnostic criteria are the ones proposed by the North American-European Consensus Conference (NAECC) in 1994,^3^ and the present study was conducted in accordance with these criteria. These criteria are comprised of onset (acute and persistent), oxygenation (the ratio of partial pressure arterial oxygen to the fraction of inspired oxygen [PaO_2_/FiO_2_] <300 mmHg for acute lung injury [ALI] and PaO_2_/FiO_2 _<200 mmHg for ARDS), radiology (bilateral opacities consistent with pulmonary edema), and exclusion criteria (pulmonary artery occlusion pressure >18 mmHg and clinical evidence of left atrial hypertension). The new classification of ARDS was released in 2017; however, as the majority of the available data on morbidity and mortality are based on the old classification, we drew upon the latter as the cutoff point for the definition of hypoxemia in the current study.^4^ The clinical conditions that can cause ARDS are divided into 2 groups of direct and indirect mechanisms, with each group including common and less common conditions. According to this classification, ARDS in coronary artery bypass grafting (CABG) is one of the indirect and less common mechanisms, although the mortality and morbidity rate of about 50% makes it an important complication in cardiac surgery.^5^^, ^^6^

The activation of the inflammatory cascade during CABG is a prominent event which could potentially affect all organ and systems, including the lung and the pulmonary function.^7^^, ^^8^ The impact of the activation of the inflammatory cascade in terms of pulmonary damage following cardiopulmonary bypass (CPB) can be studied in functional, histological, physiological, and biochemical categories. The physiological changes are mainly an abnormal exchange of gases and poor pulmonary mechanisms following CPB due to a rise in pulmonary permeability, pulmonary vascular resistance, and variations in surfactant quantities.^9^^-^^11^ Sometimes the differentiation of these changes from infectious or cardiogenic pathologies is challenging.^11^ Several biochemical changes can explain pulmonary damage following CPB, including neutrophil elastase, which can directly and indirectly cause damage. On the other hand, there are some chemicals released from a damaged pulmonary tissue such as 7S protein from type IV collagen breakdown and procalcitonin.^12^^, ^^13^ Furthermore, following CPB, the activation of the inflammatory cascade, including polymorphonuclear leukocyte activation and cytokine activation (i.e., interleukins 1, 2, 6, and 8 and tumor necrosis factor-alpha), could cause serious pulmonary damage.^14^^-^^16^ The histological changes following CPB include alveolar edema, congested alveolar capillary, and the accumulation of erythrocytes and neutrophils.^17^

Imaging with computed tomography scan shows that all patients following general anesthesia are liable to experience some degree of atelectasis.^18^ Nonetheless, in CPB, there is a considerable likelihood of damage because of inflammatory activation.^19^ Several strategies have been applied to decrease pulmonary damage like the use of off-pump CABG in repeated surgeries, the prescription of corticosteroids^19^ and aspirin before CABG,^20^ normothermic CPB instead of hypothermic one,^21^ off-pump CABG,^22^^, ^^23^ the continuation of the pulmonary artery perfusion during CPB,^24^^-^^27^ and the open lung concept.^28^^, ^^29^

 Quartine et al.^30^ conducted a challenging study on the basis of the criteria of the NAECC for the diagnosis of ARDS/ALI on 474 patients in 2003 and reported interesting findings. They found a subgroup of hypoxic patients with no history of cardiac disease that, based on the NAECC criteria, were classified as ARDS/ALI patients but were treated in a non-intensive care unit (ICU) setting. These patients were attended properly in an isolated ward without mechanical ventilation. Some of these patients were transferred to the ICU and placed on mechanical ventilation for any reasons during the first 90 days. Interestingly, the mortality rate in the group staying out of the ICU was less than that of the group transferred to the ICU with the same condition. Therefore, it appears that these patients either presented a milder form of ARDS/ALI or other diseases. On the other hand, these criteria cannot essentially predict the presence of diffuse alveolar damage since there is a wide variety of reasons for the formation of non-cardiogenic pulmonary edema. In one study, 60% of the patients that were classified as ARDS/ALI based on the NAECC criteria did not have any findings of diffuse alveolar damage in the lung biopsy.^31^ Further, patients who develop diffuse alveolar damage during the course of their disease do not present it as an acute process.

As much as several studies have tried to determine the risk factors for ARDS/ALI development, it appears that the risk factors, prevalence, and actual importance of pure hypoxemia in post- CABG patients have yet to be defined. Accordingly, we designed the present study to determine the risk factors and prevalence of hypoxemia in on-pump CABG patients in the first postoperative days.

## Methods

This cross-sectional study assessed 257 patients in terms of their important arterial blood gas (ABG) variables. All the patients were scheduled to undergo CABG under CPB in Tehran Heart Center for 2 consecutive months in 2012. 

The inclusion criterion was candidacy for CABG without other concurrent surgeries or/and intubation in the operating room. The exclusion criteria comprised valvular heart diseases requiring surgery, pneumonia, hemodynamic instability prior to surgery, aortic balloon pump prior to surgery, respiratory distress and frank respiratory problems, elective or emergent intubation before surgery, a partial pressure of carbon dioxide (PaCO_2_) level of greater than 45 mmHg, a partial pressure of oxygen (PaO_2_) level of less than 60 mmHg, a forced expiratory volume in the first second (FEV1) of less than 60% or an FEV1/forced vital capacity (FVC) ratio of less than 60%, an ejection fraction of less than 30%, any need for other surgeries before extubation, neurological complications along with orientation disturbance or neurological deficit, and a body mass index of higher than 40 kg/m^2^. Twenty-five patients met the exclusion criteria and were, therefore, excluded from the study.

For all the enrolled patients, several ABGs were checked for up to 4 hours after extubation and those with hypoxemia, based on the 1994 global criteria on ABG findings, were determined. ABGs were obtained from all the patients at 1 hour after ICU admission, before extubation, and at 4 hours after extubation. Hypoxemia was considered at ARDS or ALI levels. The patients who had either a PaO_2_ level of less than 60% or an O_2_ saturation level of less than 92%, a drop of more than 30% in PaO_2_, hemodynamic instability, or respiratory distress and dyspnea had further ABGs for up to 48 hours after extubation. 

Additionally, other independent variables such as age, sex, height, weight, the body mass index (BMI), the ejection fraction and the creatinine level prior to surgery, smoking and opioid dependence, the number of grafts, the time duration of being on pump, the cross-clamp time, intubation in the ICU, ICU care, and spirometry findings were recorded. 

All the essential data were collected prospectively, with the patients’ informed consent, to be used later for research purposes. For this study, an appropriate questionnaire was designed and filled out based on the patients’ charts by trained nurses. The collected data were processed and numerically analyzed with the SPSS software, version 20.0 (Armonk, NY: IBM Corp) in the form of code sheets and master sheets. The qualitative variables were expressed as frequencies, and the quantitative variables were presented as mean, ranges, and standard deviations. The continuous variables were compared between the hypoxemic patients at the 2 previously described different cutoff points using the Student *t*-test. The categorical variables were compared between the mentioned groups using the χ^2^ test. The variables with P values of less than 0.2 in the between-group comparisons were entered into a multivariable model. A backward stepwise logistic regression model and an entry and removal probability of 0.1 were employed to determine the multiple predictors of hypoxemia. Confidence intervals of 95% and P values of less than 0.05% were reported. 

## Results

Out of a total of 257 patients, 25 were excluded from the study due to incomplete data or some exclusion criteria and, ultimately, the data on 232 patients were analyzed. Female patients accounted for 25.0% (58 cases) and male patients comprised 75.0% (174 cases) of the study population. As regards the patients’ past history, the frequencies of diabetes mellitus (n=70 [30.3%]), a history of chronic obstructive pulmonary disease (COPD) with a normal spirometry and oxygenation before surgery (n=8 [3.5%]), smoking (n=88 [38.1%]), and opioid use (n=35 [15.2%]) were reported.

The mean of age, weight, the BMI, and height was 60.60±9.42 years, 72.97±11.62 kg, 27.15±3.93 kg/m^2^, and 164.06±10.17 cm, respectively. The mean of the pump time, the cross-clamp time, the creatinine level, the ejection fraction, and the FEV1/FVC ratio was 68.09±24.30 minutes, 41.07±14.48 minutes, 1.25±0.38 mg/dL, 48.56±8.42%, and 88.56±9.49%, correspondingly. The mean time of intubation was 11.73±13.46 hours, and the mean time of ICU stay was 40.59±31.54 hours. There was no mortality during the current admission.

Table 1 illustrates the mean values of the ABG parameters at 1 hour after ICU admission, before extubation, and at 4 hours after extubation in all the patients. It also presents a comparison of these mean values with the values of the hypoxemic patients at 12 hours and 48 hours after extubation.

It is clearly seen that the mean PaO_2_/FiO_2_ ratio at all the measured times was classified as ALI. According to Table 1, in the hypoxemic patients at 12 hours and 48 hours after extubation, the mean quantities of PaO_2_/FiO_2_ were considerably low.

Table 2 demonstrates the frequency and the percentage of the patients with hypoxemia in the range of ALI or ARDS based on the value of PaO_2_/FiO_2_ (ALI: PaO_2_/FiO_2_ <300 mmHg and ARDS: PaO_2_/FiO_2_<200 mmHg).

Table 3 represents the P values of the probable confounding factors in the patients with or without hypoxemia based on the selected cutoff points.

**Table 1:** Arterial blood gas results of the patients in different measurements

	1h after ICU Admission	Before Extubation	4h after Extubation	12h after Extubation	48h after Extubation
pH	7.37±0.08	7.35±0.05	7.36±0.04	7.39±0.05	7.43±0.04
PaO_2_ (mmHg)	133.78±39.15	113.92±27.04	95.20±23.23	85.13±21.34	74.86±20.11
PaCO_2 _(mmHg)	33.68±5.98	36.72±4.56	36.98±4.80	34.91±4.46	36.00±6.85
BE (nmol/L)	-4.84±3.22	-4.74±3.10	-7.86±5.46	-2.41±3.46	-0.65±2.82
SpO_2_ (%)	98.00±1.49	97.21±1.16	96.28±1.85	95.58±2.42	93.88±2.86
PaO_2_/FiO_2_ (mmHg)	266.11±80.51	260.24±63.36	233.97 60.84	207.44 59.32	170.55±68.05

ICU, Intensive care unit; pH, Potential of hydrogen; PaO2, Partial pressure of arterial oxygen; PaCO2, Partial pressure of arterial carbon dioxide; BE, Base excess; SpO2, Blood oxygen saturation; PaO2/FiO2, Fraction of partial pressure arterial oxygen to fraction of inspired oxygen

**Table 2:** Hypoxemia prevalence in the different measurements

	1h after ICU Admission n (%)	Before Extubation n (%)	4h after Extubation n (%)	12h after Extubation n (%)	48h after Extubation n (%)
Hypoxemia in the range of ALI or PaO_2_/ FiO_2 _<300 mmHg	147 (66.5)	151 (72.2)	154 (86.0)	100 (90.9)	18 (94.7)
Hypoxemia in the range of ARDS or PaO_2_/FiO_2 _<200 mmHg	46 (20.8)	37 (17.7)	54 (30.2)	57 (51.8)	15 (78.9)

ICU, Intensive care unit; ALI, Acute lung injury; ARDS, Acute respiratory distress syndrome

**Table 3:** P values for the comparison of the probable confounding factors in the patients with or without hypoxemia in the different measurements

	1h after ICU Admission		Before Extubation		4h after Extubation		12h after Extubation		48h after Extubation	
	ALI	ARDS	ALI	ARDS	ALI	ARDS	ALI	ARDS	ALI	ARDS
Sex	0.461	0.076	0.483	0.551	0.153	0.390	0.483	0.647	0.211	0.999
DM	0.445	0.997	0.272	0.346	0.103	0.180	0.281	0.999	0.368	0.117
COPD	0.721	0.674	0.448	0.629	0.999	0.999	0.999	0.109	0.999	0.999
Smoking	0.364	0.574	0.136	0.487	0.669	0.531	0.322	0.773	0.999	0.035
Opioid	0.968	0.676	0.611	0.391	0.999	0.989	0.999	0.530	0.999	0.386
Graft number	0.736	0.559	0.679	0.410	0.632	0.567	0.039	0.566	0.468	0.029

ICU, Intensive care unit; ALI, Acute lung injury; ARDS, Acute respiratory distress syndrome; DM, Diabetes mellitus; COPD, Chronic obstructive lung disease

As can be seen in the table, there was no significant relationship between the patients’ sex, diabetes mellitus, COPD, smoking, opioid use status, and hypoxemia at all the measured times. However, the number of grafts showed a significant relationship with ALI and ARDS in the hypoxemic group at 12 and 48 hours after extubation. 

Other parameters such as age, weight, height, the creatinine level, the cross-clamp time, the pump time, the ejection fraction, and the FEV1/FVC ratio were studied in terms of the P value of their connection to hypoxemia in the range of ALI or ARDS at the mentioned times. According to the findings and based on ANOVA, there were no significant relationships between height, age, the intubation time, the pump time, the ejection fraction, the FEV1/FVC ratio, ICU care time, and hypoxemia in the range of ALI or ARDS. The relationship between weight and hypoxemia in the range of ALI showed a P value of 0.001 at 1 hour after ICU admission and at 4 hours after extubation. The noticeable point is that there was no association between weight and hypoxemia in the range of ALI in the hypoxemic group. A rise in the cross-clamp time showed a significant relationship with the risk of hypoxemia in the range of ARDS at 4 hours after extubation (P=0.014). This relationship was not detected in the hypoxemic group. The same trend was seen between the rise in the patients’ weight and hypoxemia in the range of ARDS at 1 hour after ICU admission, before extubation, and at 4 hours after it.

On the other hand, a rise in the creatinine level showed an association with a rise in the risk of hypoxemia in the range of ARDS at 12 hours after extubation in the hypoxemic patients.

In this study, repeated measurement analysis was used to determine the factors affecting the mean PaO_2_/FiO_2_ ratio, and the effect of each factor is reported based on the P value. The results revealed that in the entire study population at 1 hour after ICU admission, before extubation, and at 4 hours after extubation, only an increase in the weight of the patients had an effect on the PaO_2_/FiO_2 _ratio. In the selected group of patients with persistent hypoxemia at 12 and 48 hours after extubation, the PaO_2_/FiO_2_ ratio was influenced by the pump time and the COPD history only.

## Discussion

The purpose of the present study was to determine the prevalence and predictive factors of oxygenation disturbance at different time periods following CABG under CPB. Additionally, the effects of acute hypoxemia on the short-term prognosis including intubation duration, ICU length of stay, and mortality were studied. In this study, due to an inadequacy of facilities, the only criterion for the inclusion of the patients in the ARDS or ALI class was the PaO_2_/FiO_2 _ratio.

According to a study conducted by Milot et al.^5^ from 1995 until 1997 on 3278 patients undergoing CABG under CPB, the most important independent predictive factors to cause ARDS were a past history of cardiac surgery, frequent blood product transfusion, and shock. In our study, we excluded patients with a past history of cardiac surgery and did not study transfusion as a factor. Additionally, we removed the risk of shock by assessing the use of inotropes and balloon pumps. 

While in the study by Milot et al.^5^ and an older one by Christenson et al.,^32^ the occurrence rate of ARDS and its mortality rate were reported to be 15% and 0.04% and 68.4% and 1%, respectively, in our study, the mortality rate in the patients with hypoxemia in the range of ARDS was considerably low (0% during ICU care). This can be attributed to the exclusion of patients with a history of cardiopulmonary disease from the study. In other studies, the infusion of pumped blood volumes (>300 L) has been considered to be a predisposing factor to ARDS formation in CABG patients.^6^ Our findings showed that the decrease in the mean PaO_2_/FiO_2_ ratio had a significant relationship with an increase in the pump time only in the group of hypoxemic patients. This proves that although the rise in the pump time did not affect the PaO_2_/FiO_2_ ratio during the early hours, it did exert a delayed effect on the hypoxemic patients (at 12 and 48 h after extubation). This could be related to the systemic inflammatory process induced by the CPB pump, even after extubation. Similarly, a past history of COPD with a normal spirometry and oxygenation affected the mean PaO_2_/FiO_2_ ratio only in the group of hypoxemic patients after CABG (at 12 and 48 h after surgery). This demonstrates that even a controlled COPD can play a role in patients’ hypoxemia, at least in the delayed phase.

We found a significant relationship between an increase in the number of grafts and a rise in the risk of hypoxemia during the first studied time periods. Additionally, there was a significant relationship between PaO_2_/FiO_2_ in ARDS, the ALI range, and obesity and the BMI, particularly early in the study; nevertheless, this effect was temporary and after some time, these patients recovered from the primary hypoxemic state. The considerable impact of these 2 factors during the first hours can be attributed to this population’s specific physiology and the higher retention of anesthetic and narcotic medications. Such patients will most probably recover from the hypoxic state following the recovery of their pulmonary function.

In our study, some possible confounders such as smoking history did not show a statistically meaningfully higher coincidence in the hypoxemic patients, although smoking cessation has been previously shown to decrease hypoxemia and even reduce ICU stay in on-pump post-CABG patients.^33^ Considering the high prevalence of hypoxemia and its critical role in the establishment of ARDS diagnosis, this hypoxemia appears to be a benign and self-limited phenomenon by comparison with its association with ARDS. This could affect the accuracy of the ARDS criteria. It would, therefore, be advisable to revise and adjust the ARDS criteria for cardiac surgery patients.

In the present cross-sectional and single-center study, we tried to eliminate the effects of confounders, but the complexity and characteristics of the disease render it impossible in such studies. Furthermore, the necessity of pulmonary catheter implantation and wedge pressure assessments in our patients precluded us from ruling out cardiogenic pulmonary edema and we were unable to assume the prevalence of hypoxemia as the exact prevalence of ALI and ARDS in our CABG patients. Still, we did try to minimize the interference of cardiogenic hypoxemia by excluding patients with echocardiographic or hemodynamic evidence of cardiogenic hypoxemia, even if mixed pathologies were possible. Another salient weakness is that we failed to consider some possible confounders such as high levels of serum free fatty acids, which have recently been described as risk factors for the development of hypoxemia in on-pump CABG patients.^34^

## Conclusion

The prevalence of hypoxemia in on-pump CABG patients is very common, and it could be persistent for up to 48 hours after extubation. Be that as it may, this hypoxemia is a benign event without mortality. This high prevalence and also the benign nature of this variety of hypoxemia could challenge the accuracy and positive or negative predictive value of the routine ARDS criteria.

## Notes:

***This paper should be cited as: ***Yousefshahi F, Samadi E, Paknejad O, Movafegh A, Barkhordari K, Bastan Hagh E, Dehestani B. Prevalence and Risk Factors of Hypoxemia after Coronary Artery Bypass Grafting: The Time to Change Our Conceptions. J Teh Univ Heart Ctr 2019;14(2):74-80.
